# Spinal Tuberculosis in a Young Male Patient With Empty Sella Syndrome and Panhypopituitarism

**DOI:** 10.7759/cureus.78223

**Published:** 2025-01-30

**Authors:** Xin Wei Choo, Jing Wen Wong, Mohd Arif Abdul Malik Khiew, Kai Siang Khoo, Anusha Ramanaidu, Anweeitha Ramasamy, Chee Yik Chang

**Affiliations:** 1 Internal Medicine, Sultanah Aminah Hospital, Johor Bahru, MYS; 2 Radiology, Sultanah Aminah Hospital, Johor Bahru, MYS; 3 Pathology, Sultanah Aminah Hospital, Johor Bahru, MYS; 4 Infectious Diseases, Sultanah Aminah Hospital, Johor Bahru, MYS

**Keywords:** empty sella, mycobacterium tuberculosis, panhypopituitarism, pott’s disease-tuberculous spondylitis, spinal tuberculosis

## Abstract

Spinal tuberculosis (TB), or Pott's disease, is a rare but serious form of extrapulmonary TB that primarily affects the thoracic spine and can result in severe neurological complications. Patients with underlying endocrine disorders, such as panhypopituitarism, are at increased risk of developing infections due to immune suppression caused by hormonal deficiencies and long-term steroid replacement therapy. We report the case of a 24-year-old Malay male patient with a known history of empty sella syndrome and panhypopituitarism on hormone replacement therapy, who presented with progressive left lower limb weakness over one week. An MRI of the spine revealed a multiloculated pre- and paravertebral collection with intraspinal extension. A spine biopsy confirmed the presence of *Mycobacterium tuberculosis* via TB GeneXpert testing. The patient was started on anti-TB therapy. However, due to worsening neurological function, he underwent posterior spinal fusion and decompression surgery, which resulted in improved lower limb function. Spinal TB should be considered in patients with neurological deficits and predisposing conditions, such as endocrine disorders, even in the absence of classical TB symptoms.

## Introduction

Spinal tuberculosis (TB), also known as Pott's disease, is a form of extrapulmonary TB that can lead to significant morbidity, particularly in immunocompromised individuals. The thoracic spine is most commonly affected, and complications such as vertebral body destruction, spinal instability, and neurological deficits can occur if the condition is not diagnosed and treated promptly [1,2].

Patients with endocrine disorders such as diabetes mellitus, primary adrenal insufficiency, hypercortisolism, and panhypopituitarism are at an increased risk of infections due to immune suppression, which is further exacerbated by long-term steroid replacement therapy [3]. Herein, we present the case of a young man with empty sella syndrome and panhypopituitarism who developed spinal TB, presenting with progressive lower limb weakness, and outline the management approach.

## Case presentation

A 24-year-old Malay man, formerly employed as an assistant manager at a supermarket, presented with a one-week history of progressive left lower limb weakness, which had worsened and extended proximally to his thigh over the preceding two days. His medical history was significant for empty sella syndrome with panhypopituitarism, managed with hydrocortisone and thyroxine replacement therapy. Over the past year, he had experienced significant weight loss of approximately 15 kg and a marked reduction in appetite. He denied symptoms suggestive of infection, such as fever, respiratory symptoms, or exposure to TB, as well as gastrointestinal disturbances or urinary incontinence.

On physical examination, he was alert and oriented, appearing to be in good general condition. Examination of the lungs and cardiovascular system was unremarkable, with no palpable lymph nodes. A neurological assessment revealed marked weakness in the left lower limb, with muscle power graded at 1/5 (MRC scale), while the right lower limb demonstrated normal strength. Babinski reflexes were upgoing bilaterally, and hyperreflexia was noted. Tenderness was present over the thoracic spine, and sensation was diminished up to the T8 dermatome. Examination of the cranial nerves and anal tone was normal.

**Figure 1:**
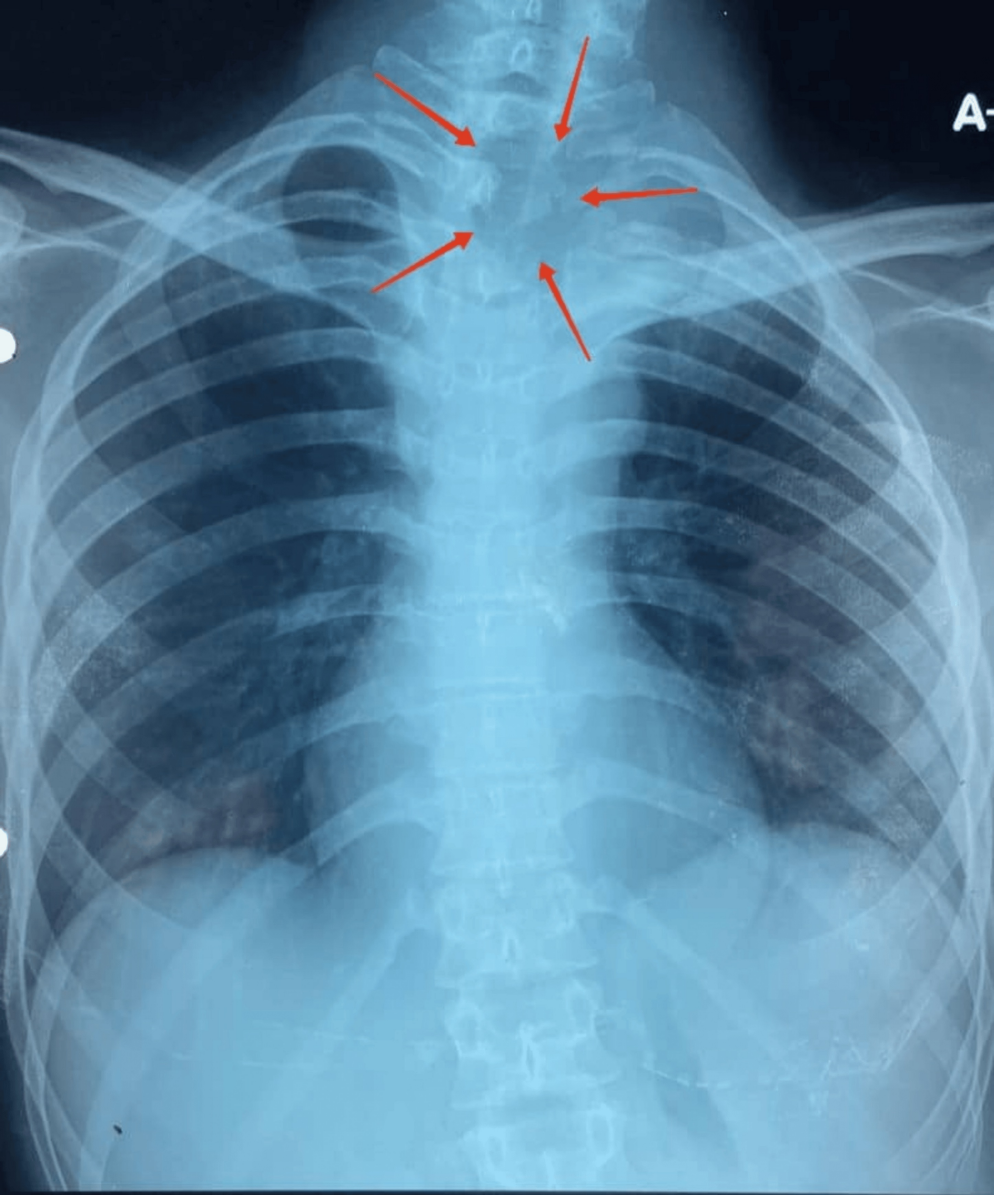
Chest radiography shows lytic bony destruction of the body of T2 and T3 vertebrae involving the respective left pedicles and transverse process with surrounding opacities

**Figure 2:**
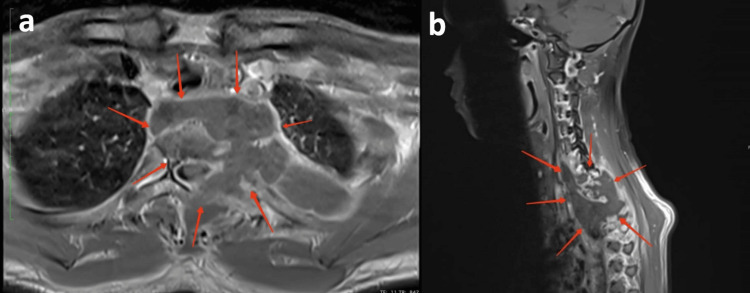
(a) T1WI in axial view post-contrast (at the level of T2 vertebrae) shows a multiloculated rim-enhancing collection, (b) T1WI in sagittal view post-contrast (at the cervicothoracic spine involving C7 to T5), shows a multiloculated collection involving the prevertebral and paravertebral region with subligamentous spread.

**Figure 3:**
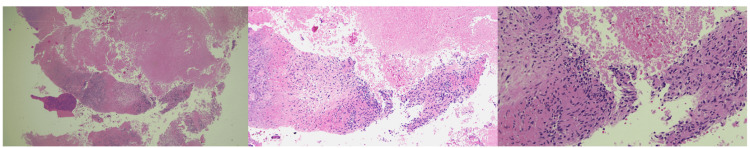
Histopathological examination of the spinal tissue biopsy showing presence of granuloma in the power of 4, 10, 20.

Chest radiography showed lytic bony destruction of the body of T2 and T3 vertebrae involving the respective left pedicles and transverse process with surrounding opacities (Figure 1). Magnetic resonance imaging (MRI) of the entire spine revealed a multiloculated pre- and paravertebral rim-enhancing collection extending from the C7/T1 level to the T4/5 level, measuring 2.8 × 9.6 × 7.5 cm. There was intraspinal extension into the epidural space at the left T2/3 level (Figure 2). A spine biopsy was performed, and histopathological examination revealed necrotizing granulomatous inflammation (Figure 3). Subsequent TB GeneXpert MTB/RIF assay testing and mycobacterial culture confirmed *Mycobacterium tuberculosis, *which was sensitive to all first-line anti-tuberculous drugs*.* Anti-tuberculous therapy with oral Akurit-4 (four tablets daily) was initiated. At the time of discharge, the patient’s neurological status remained unchanged. He was scheduled for follow-up care under the respiratory and orthopedic teams.

During follow-up with the orthopedic team after two weeks of anti-TB therapy initiation, his neurological deficits persisted, including left lower limb hypertonia, persistent hyperreflexia, and no improvement in muscle power. Given the lack of response to ongoing anti-TB therapy, surgical intervention was deemed necessary. The patient underwent posterior spinal fusion (PSF) from C5 to T7 and decompression from T2 to T4. Postoperatively, the patient demonstrated improvement in left lower limb function three weeks after surgery, with muscle power increasing to 3/5 on the MRC scale. He was continued on anti-TB therapy, which was maintained through follow-up care with the respiratory and orthopedic teams. 

## Discussion

TB is a potentially life-threatening complication of immunosuppressive therapy. Beyond its effects on humoral immunity, corticosteroids impair cellular immunity, which serves as the primary defense against TB. Spinal TB typically arises from the hematogenous dissemination of *Mycobacterium tuberculosis* from the pulmonary or genitourinary systems to the richly vascularized vertebral bodies [4]. The disease often has an insidious onset, presenting with nonspecific symptoms such as malaise, weight loss, appetite loss, and night sweats. Back pain, which is commonly localized to the thoracic spine, is the most common symptom of spinal TB, but it was not present in this case [5].

Endocrine dysfunctions, such as panhypopituitarism and adrenal insufficiency, are often overlooked risk factors for TB. These conditions impair both cellular and humoral immune responses, thereby increasing susceptibility to TB [6]. In this case, the patient’s underlying empty sella syndrome and panhypopituitarism likely contributed to the development and dissemination of spinal TB. Adrenal insufficiency, a common feature of panhypopituitarism, leads to cortisol deficiency, which severely impairs the immune system. Cortisol plays a key role in modulating the immune response by maintaining cytokine balance and enhancing macrophage activation, both of which are critical in controlling *Mycobacterium tuberculosis* [7]. The lack of cortisol reduces the ability to activate a strong immune defense, increasing the risk of TB infection and progression. This results in an increased incidence of TB in patients with endocrine disorders. Furthermore, regions with endemic TB such as Southeast Asia, Sub-Saharan Africa, and Eastern Europe often have higher rates of delayed diagnosis in patients with coexisting endocrine conditions, leading to severe manifestations such as disseminated TB [8].

The diagnosis of spinal TB is a form of extrapulmonary TB, which usually presents with nonspecific clinical presentation and frequent absence of pulmonary involvement. Extrapulmonary TB, particularly spinal TB, requires more invasive approaches to confirm the disease compared to pulmonary TB in which sputum analysis and imaging are often able to provide definitive clues. Histopathological evaluation through tissue biopsy is the gold standard for diagnosing spinal TB as it provides definitive evidence by showing granulomatous inflammation with caseating necrosis and/or the presence of *Mycobacterium tuberculosis* [9]. Biopsy is crucial as it allows for culture and drug susceptibility testing. Imaging modalities such as MRI can suggest spinal TB through findings like vertebral destruction, paravertebral abscesses, or spinal deformities, although these are not pathognomonic. Neuroimaging-guided needle biopsy allows precise sampling of the affected vertebral body or paravertebral tissues, even in challenging locations as it minimizes procedural risks while ensuring diagnostic accuracy [10]. In cases with atypical presentations or equivocal imaging findings, a biopsy can provide the diagnostic clarity needed for appropriate management. In this patient, disseminated TB involved the spine without pulmonary symptoms or typical findings on thoracic radiography, emphasizing the importance of tissue biopsy as it enables prompt initiation of anti-TB therapy and prevents further complications.

Anti-TB therapy remains the mainstay of treatment of spinal TB, with surgery reserved for specific indications. The decision between conservative management and surgical intervention depends on the severity of the disease, the presence of complications, and the patient’s clinical status. Patients with no or minimal neurological symptoms, no evidence of significant vertebral destruction or instability, resolution of symptoms, and imaging findings with anti-TB therapy alone can often be treated medically [11]. Surgery is indicated when medical management alone is insufficient to manage complications or when the disease progresses despite adequate anti-TB therapy. Surgery is also required in cases of worsening or severe neurological impairment caused by spinal cord or nerve root compression. Neurological deficits, reported in 23-76% of cases, are also frequent but rarely constitute the initial presentation [12]. In active disease, the primary mechanisms underlying Pott’s paraplegia include spinal cord compression by abscesses, necrotic debris, tuberculomas, or sequestrum of bone or disk material. Vertebral column destruction can further lead to instability, resulting in subluxation or dislocation of the spine. The primary goals of surgery in spinal TB are to relieve spinal cord or nerve root compression caused by abscesses, debris, or vertebral fragments and to restore spinal stability using implants, bone grafts, or fusion techniques [13]. 

While anti-TB therapy effectively reduces the infectious load and resolves abscesses, it does not report structural damage caused by TB. The persistent instability in this patient’s spine likely served as a continuous source of neural compromise, interfering with the therapeutic effects of anti-TB therapy. Neurological deficits caused by such mechanical factors often require surgical intervention for resolution. Spinal instability is a key contributor to neurological symptoms in spinal TB. Vertebral destruction, deformity, or collapse can result in mechanical compression of the spinal cord or nerve roots, either directly through bony fragments or indirectly by altering spinal alignment. The dynamic nature of instability exacerbates compression during movement, affecting nerve function and delaying recovery despite effective anti-TB therapy [14]. In this patient, the structural instability likely prevented the adequate decompression of the spinal cord, even as anti-TB therapy reduced infection and inflammation.

## Conclusions

Early diagnosis of spinal TB is difficult due to its nonspecific clinical presentation. Panhypopituitarism could potentially increase the risk of tuberculosis infection. MRI is the preferred diagnostic imaging modality and frequently shows paravertebral collections, cold abscesses, vertebral collapse, and spinal deformities. For definitive diagnosis, neuroimaging-guided needle biopsy of the affected vertebral body remains the gold standard for histopathological evaluation. Early intervention is crucial in improving patient outcomes.
